# Zuonin B Inhibits Lipopolysaccharide-Induced Inflammation via Downregulation of the ERK1/2 and JNK Pathways in RAW264.7 Macrophages

**DOI:** 10.1155/2012/728196

**Published:** 2012-02-01

**Authors:** Mee-Young Lee, Ji-Eun Yuk, Ok-Kyung Kwon, Sei-Ryang Oh, Hyeong-Kyu Lee, Kyung-Seop Ahn

**Affiliations:** ^1^Immune Modulator Research Center, Korea Research Institute of Bioscience and Biotechnology, 685-1 Yangchung-ri, Ochang-eup, Cheongwon-gun, Chungbuk, Republic of Korea; ^2^Herbal Medicine, Evidance Based Medicine Research Center, Korea Institute of Oriental Medicine, Exporo 483, Yusung, Daejeon 305-811, Republic of Korea

## Abstract

We investigated whether Zuonin B exerts immunological effects on RAW264.7 cells. Zuonin B, isolated from flower buds of *Daphne genkwa*, suppressed the levels of nitric oxide and prostaglandin E_2_, as well as proinflammatory cytokines, such as tumor necrosis factor-**α** and interleukin-(IL-) 6, in lipopolysaccharide-stimulated macrophages. Moreover, the compound inhibited cyclooxygenase-2 and inducible nitric oxide synthase expression. Zuonin B attenuated NF-kappaB (NF-**κ**B) activation via suppressing proteolysis of inhibitor kappa B-alpha (I**κ**B-**α**) and p65 nuclear translocation as well as phosphorylation of extracellular signal-regulated kinase 1/2 and c-Jun N-terminal kinase. Additionally, IL-4 and IL-13 production in ConA-induced splenocytes was inhibited by Zuonin B. In conclusion, the anti-inflammatory effects of Zuonin B are attributable to the suppression of proinflammatory cytokines and mediators via blockage of NF-**κ**B and AP-1 activation. Based on these findings, we propose that Zuonin B is potentially an effective functional chemical candidate for the prevention of inflammatory diseases.

## 1. Introduction

Inflammation is a multistep process mediated by activated inflammatory and immune cells, including macrophages and monocytes [1], and comprises a complex series of reactions regulated by a cascade of cytokines, growth factors, nitric oxide (NO), and prostaglandins (PGs) produced by active macrophages. Macrophages are key players in the immune response to foreign invaders, such as proinflammatory cytokines [2]. We made the highlighted change to the second address. 

NO, a reactive radical produced from the guanidino nitrogen of l-arginine by NO synthase (NOS), is essential for host innate immune responses to pathogenic bacteria, viruses, fungi, and parasites [3]. However, excessive NO production can result in the development of inflammatory diseases, including rheumatoid arthritis and autoimmune disorders [4]. PGE_2_ is an inflammatory mediator produced during the conversion of arachidonic acid by cyclooxygenase. In various inflammatory cells, COX-2 is induced by cytokines and other activators, such as LPS, resulting in the release of a large amount of PGE_2_ at the inflammatory sites [5]. Cytokines are produced and secreted by a variety of cell types, including macrophages and monocytes. These proteins play a major role in the induction and regulation of cellular interactions (e.g., inflammation, hematopoiesis, allergy, and immunoreaction) [6].

Nuclear transcription factor kappa-B (NF-*κ*B) regulates various genes involved in immune and acute phase inflammatory responses as well as cell survival [7]. NF-*κ*B activation in response to proinflammatory stimuli involves the rapid phosphorylation of I*κ*Bs by the IKK signalosome complex [8]. The resulting free NF-*κ*Btranslocates to the nucleus, where it binds to NF-*κ*B-binding sites in the promoter regions of target genes and induces the transcription of proinflammatory mediators, such as iNOS and COX-2. In addition to NF-*κ*B, mitogen-activated protein kinases (MAPKs) are implicated in cytokine production in macrophages [9]. Three MAPK families (extracellular signal-regulated kinase (ERK)1/2, p38, and c-Jun N-terminal kinase (JNK)) are signaling molecules that react to extracellular stimuli (mitogens) and regulate immune responses, including proinflammatory cytokine production, mitosis, differentiation, and cell survival/apoptosis [9, 10]. A major consequence of MAPK phosphorylation is activation of these transcription factors, which serve as immediate or downstream substrates of the kinases [11].

In a previous study, we isolated nine lignans from the dried flower buds of *Machilus thunbergii*, specifically, machilin A, licarin B, Zuonin B, macelignan, oleiferin C, *meso*-dihydroguaiaretic acid, licarin A, machilin F, and nectandrin B [12]. The molecular mechanism and activity of Zuonin B in macrophages remain to be clarified. To establish the mechanisms underlying the anti-inflammatory effects of Zuonin B, in the present study we investigated the expression patterns of inflammatory mediators in LPS-stimulated RAW264.7 cells. Additionally, we examined the effects of Zuonin B on MAPK and NF-*κ*B activation.

## 2. Materials and Methods

### 2.1. Extraction and Isolation of Zuonin B

The stem bark of *M. thunbergii *(1.8 kg) was treated with MeOH at room temperature to produce a dark brown extract (290 g). The MeOH extract was suspended in H_2_O, and extracted with hexane to produce a hexane-soluble fraction (35.3 g). The hexane-soluble fraction was subjected to chromatography on a silica gel column (500 g) and eluted using a gradient of hexane and acetone to yield four fractions. Repeated column chromatography of Fr. 2 (10.9 g) on a silica gel (hexane/acetone; 7 : 3 and benzene/EtOAc; 20 : 1) and ODS column (MeOH, 10% aq. MeOH) afforded Zuonin B(75 mg). The Zuonin B was purified as colorless needles with the following characteristics: mp 49–51°C; [*α*]_*D*_ 0°(*c* = 0.33, CHCl_3_); UV*λ*_max⁡_ (CHCl_3_) nm (log⁡_*ε*_): 294 (2.45); FAB-MS *m*/*z*: 363 [M+Na]^+^.

### 2.2. Cell Culture

The RAW264.7 cell line derived from murine macrophages was obtained from the American Type Culture Collection (ATCC, Rockville, MD, USA). Cells were maintained in Dulbecco's modified Eagle's medium supplemented with glutamine (1 mM), 10% heat-inactivated fetal bovine serum (FBS), penicillin (50 U/mL), and streptomycin (50 *μ*g/mL) at 37°C in an atmosphere of 5% CO_2_. Cells that reached a density of 5 × 10^4^ cells/mL were activated by incubation in medium containing *E. coli* LPS (1 *μ*g/mL). LPS was added to a range of concentrations of test compounds dissolved in DMSO. Cells treated with 0.05% DMSO were used as the vehicle control.

### 2.3. MTT Assay for Cell Viability

Cells were seeded into 96-well plates at a density of 5 × 10^4^ cells/well, and incubated with serum-free medium in the presence of different concentrations of Zuonin B. After incubation for 24 h, 10 *μ*L of 3-(4,5-dimethylthiazol-2-yl)-2,5-diphenyl tetrazolium bromide (MTT) (5 mg/mL in saline) was added and incubation continued for another 4 h. Mitochondrial succinate dehydrogenase in live cells converts MTT to visible formazan crystals during incubation. Formazan crystals were solubilized in dimethylsulfoxide, and the absorbance measured at 540 nm using an enzyme-linked immunosorbent assay (ELISA) microplate reader (Benchmark, Bio-Rad Laboratories, CA, USA). The relative cell viability was calculated and compared with the absorbance of the untreated control group. All experiments were performed in triplicate.

### 2.4. Preparation and Treatment of Splenocyte Suspensions

Spleens from BALB/c mice were removed aseptically, and a single-cell suspension of splenocytes obtained by passing the cells through two needles in RPMI 1640 containing 10% fetal bovine serum, 25 mM HEPES, 2 mM glutamine, 100 U/mL penicillin, and 100 mg/mL streptomycin (GibcoBRL, NY, USA). Red blood cells (RBCs) were lysed with lysis buffer (Sigma Chemical, St Louis, MO, USA) at 37°C for 10 min. After washing with PBS, cells were cultured in 100*φ* dishes for 4 h. Splenocytes were plated into 96-well plates at a density of 1 × 10^6^ cells/mL and treated with different concentrations of *p*-hydroxycinnamic acid methyl ester for 1 h, followed by ConA (1 *μ*g/mL) for a further 3 days. The IL-4 and IL-13 levels in culture supernatants were measured using ELISA kits for murine cytokines (R&D systems, MN, USA), according to the manufacturer's instructions. All experimental procedures were carried out in accordance with the NIH Guidelines for the Care and Use of Laboratory animals, and animal handling followed the dictates of the National Animal Welfare Law of Korea. 

### 2.5. TNF-*α* and IL-6 Assays

TNF-*α* and IL-6 production in RAW264.7 cells were assayed using ELISA kits (Assay design, USA) following the manufacturer's instructions. Cells (1 × 10^6^cells/well) in 96-well plates were treated with different concentrations of Zuonin B for 1 h, production of TNF-*α* and IL-6 stimulated with 1 *μ*g/mL of LPS, and incubation continued for another 24 h. The conditioned medium was used for the subsequent experiment. Specifically, 50 *μ*L of TNF-*α* standards (prepared for calibration) or a similar volume of Zuonin-B-treated conditioned medium was added to the wells of TNF-*α* and IL-6 antibody-coated 96-well plates in triplicate. Absorbance was determined at 450 nm using the microplate reader. Specific standard curves were employed to quantify the amounts of TNF-*α* and IL-6 released by cells.

### 2.6. Measurement of Nitric Oxide (NO) Production

The nitrite concentration in culture medium was measured as an indicator of NO production, according to the Griess reaction. RAW264.7 cells (2 × 10^5^ cells/well) were cultured in 96-well plates using DMEM without phenol red and pretreated with different concentrations of Zuonin B for 1 h. Cellular NO production was induced by adding a 1 *μ*g/mL of LPS and incubating for 24 h. Next, 100 *μ*L of conditioned medium was mixed with an equivalent volume of Griess reagent and incubated for 15 min. The absorbance of the mixture at 540 nm was measured with an ELISA microplate reader (Benchmark, Bio-Rad Laboratories, CA, USA). The values obtained were compared with those of standard concentrations of sodium nitrite dissolved in DMEM, and the concentrations of nitrite in the conditioned media of sample-treated cells calculated.

### 2.7. Measurement of PGE_2_ Levels

Production of PGE_2_, one of the mediators released after activation of COX-2, was used as a marker for COX-2 assessment. RAW264.7 cells (2 × 10^5^ cells/well) were cultured in 96-well plates with serum-free medium and pretreated with different concentrations of Zuonin B for 1 h. PGE_2_ generation (via COX-2 activation) was stimulated by adding a 1 *μ*g/mL of LPS and incubating for 24 h. The conditioned medium was used for PGE_2_ determination with a prostaglandin E_2_ ELISA assay kit (Cayman Chemical Co., Ann Arbor, MI, USA), according to the manufacturer's instructions. The absorbance was measured at 450 nm using an enzyme-linked immunosorbent assay (ELISA) microplate reader (Benchmark, Bio-Rad Laboratories, CA, USA).

### 2.8. Western Blot Analysis

RAW264.7 cells exposed to Zuonin B were treated with lysis buffer containing protease inhibitors (50 mM Tris-HCl (pH 7.4), 150 mM NaCl, 1 mM EDTA, 0.5% NP-40, 0.1% SDS, 1 mM EGTA, 100 *μ*g/mL PMSF, 10 *μ*g/mL pepstatin A, and 100 *μ*M Na_3_VO_3_). Homogenates were centrifuged at 12000 g at 4°C for 25 min, and protein concentrations in the supernatant fractions determined using Bradford reagent (Bio-Rad, Hercules, CA, USA). Proteins were subjected to sodium dodecyl sulfate-polyacrylamide gel electrophoresis (SDS-PAGE) at 100 V for 90 min on 4% to 12% gradient gels. Separated proteins were transferred to polyvinylidenedifluoride (PVDF) membranes (Amersham Biosciences, Piscataway, NJ, USA). After blocking nonspecific binding sites with 5% nonfat dry milk dissolved in TBST buffer (10 mM Tris-HCl, pH 7.5, 150 mM NaCl, 0.1% Tween-20) overnight at 4°C, membranes were incubated overnight at 4°C with anti-iNOS, COX-2, ERK1/2, JNK, p65, I*κ*B-*α*, AP-1, *β*-actin, and PARP antibodies. Following removal of the primary antibody, membranes were washed three times with TBST buffer at room temperature and subsequently incubated with horseradish-peroxidase-(HRP-) conjugated secondary antibody for 1 h at room temperature. Membranes were rewashed with TBST buffer, and the immunoreactive bands visualized using ECL reagent (Amersham Pharmacia Biotech, Uppsala, Sweden).

### 2.9. Immunofluorescence Analysis

 RAW264.7 cells cultured on Permanox plastic chamber slides were fixed with ethanol for 30 min at 4°C. After washing with PBS and blocking with 3% bovine serum albumin in PBS for 30 min, samples were incubated overnight at 4°C with rabbit polyclonal anti-iNOS, anti-COX-2 (1 : 500 dilution, Santa Cruz Biotechnology, Santa Cruz, CA, USA), and anti-NF-*κ*B p65 subunit (1 : 500 dilution, Assay Designs) antibodies. Excess primary antibody was removed, slides washed with PBS, and the samples incubated with Texas Red-conjugated secondary antibody (SantaCruz Biotechnology) for 2 h at room temperature. After washing, slides were mounted using ProLong Gold Antifade reagent containing 4′,6-diamidino-2-phenylindole (DAPI) (Invitrogen, USA) to visualizethe nuclei. Specimens were covered with coverslips and evaluatedunder a confocal laser scanning microscope (LSM510m, Carl Zeiss, Germany).

### 2.10. Statistical Analysis

Data were expressed as means ± standard error of the mean (SEM). Statistical significance was determined using the ANOVA test for independent means. The critical level for significance was set at *P* < 0.05.

## 3. Results

### 3.1. Effects of Zuonin B on Macrophage Toxicity

**Figure 1:**
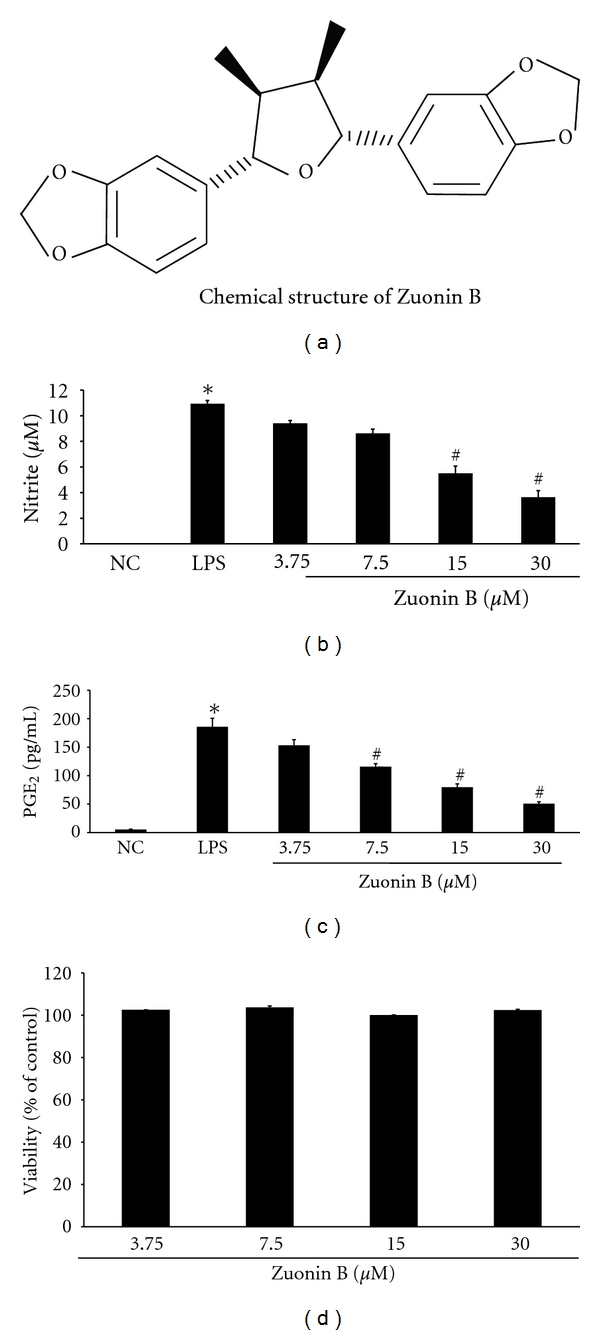
Effects of Zuonin B on inhibition of LPS-induced nitric oxide (NO) and prostaglandin E_2_ (PGE_2_) production in RAW264.7 cells. Cells cultured in phenol red and serum-free media were pretreated with different concentrations of Zuonin B for 1 h and stimulated with a 1 *μ*g/mL LPS for 24 h. (a) Structure of Zuonin B. (b) NO production. (c) Amount of PGE_2_ release. (d) Cell viability. NC: untreated control cells; LPS: LPS only treatment. Data represent mean values of three experiments ± SEM. *Significant difference from NC and ^#^significant difference from LPS, *P* < 0.05.

The MTT cell viability assay was performed using RAW264.7 cells grown in medium to determine the effects of Zuonin B (Figure 1(a)). The cytotoxic effect of Zuonin B in RAW264.7 cells was examined to establish the appropriate concentration range for analysis of COX-2 and iNOS expression. Neither Zuonin Bnor DMSO exerted a significant toxic effect on RAW264.7 cells at the concentrations 3.75, 7.5, 15, and 30 *μ*M examined after 24 h of treatment (Figure 1(d)). Thus, the nontoxic concentrations of Zuonin B were used for subsequent experiments.

### 3.2. Effects of Zuonin B on NO and PGE_2_ Production in RAW264.7 Cells

The effects of Zuonin B on LPS-induced NO production in RAW264.7 cells were investigated by estimating the amount of nitrite released into the culture medium using the Griess reaction. To ascertain whether Zuonin B inhibits LPS-induced nitrite production and iNOS protein expression, RAW264.7 cells were pretreated for 1 h with various concentrations of the compound and subsequently treated with 1 *μ*g/mL LPS. No significant differences in NO production were observed in RAW264.7 cells treated with Zuonin B alone, compared with the negative control (data not shown). As shown in Figure 1(b), Zuonin B suppressed nitrite production in a concentration-dependent manner, with >50% inhibition at a concentration of 30 *μ*M. The COX-2 levels were examined with the PGE_2_ immunoassay to determine whether Zuonin B inhibition of COX-2 production is related to modulation of PGE_2_ release. Notably, pretreatment of cells with Zuonin B markedly inhibited the LPS-induced increase in PGE_2_ production in a concentration-dependent manner (Figure 1(c)).

### 3.3. Effects of Zuonin B on iNOS and COX-2 Protein Expression in RAW264.7 Cells

**Figure 2:**
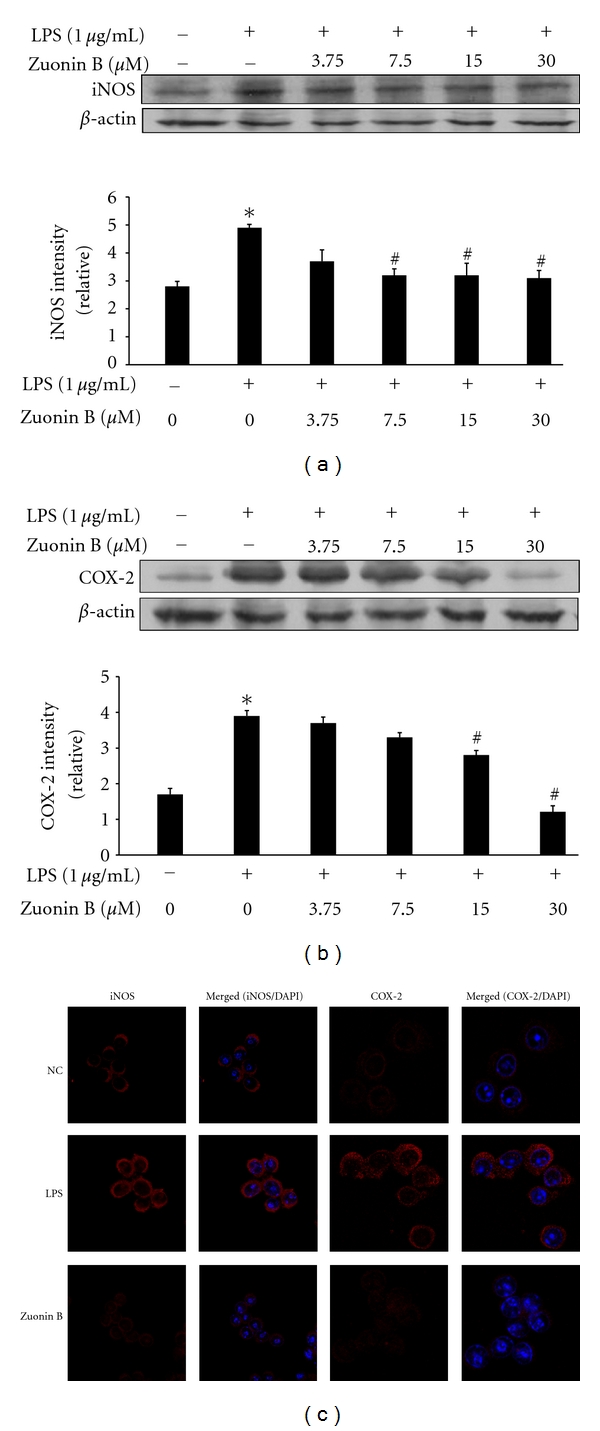
Inhibition of inducible nitric oxide synthase (iNOS) and cyclooxygenase-2 (COX-2) expression by Zuonin B. RAW264.7 cells were pretreated with different concentrations of Zuonin B for 1 h and stimulated with LPS (1 *μ*g/mL) for a further 24 h. Equal amounts of protein in cell lysates were electrophoresed, and the protein expression levels of iNOS (a) and COX-2 (b) determined using specific antibodies for iNOS and COX-2. The respective levels of *β*-actin were used to confirm equal amounts of protein loading for electrophoresis. Interleukin COX-2 and iNOS mRNA levels in lung tissue, (c) Cells were cultured for 24 h with LPS (1 *μ*g/mL), fixed, permeabilized, and incubated with rabbit polyclonal anti-iNOS and COX-2 antibody, followed by Texas-red conjugated anti-rabbit Ig (red). The nuclei of the corresponding cells were visualized with 4′,6-diamidino-2-phenylindole (DAPI) (magnification  ×400). NC: untreated control cells; LPS: LPS only treatment.

To establish the anti-inflammatory activity of Zuonin B, we tested its effects on LPS-induced iNOS and COX-2 protein upregulation in RAW264.7 cells via Western blot and immunofluorescence analyses. As shown in Figures 2(a) and 2(c), iNOS protein expression was not detected in unstimulated cells, but was markedly increased by 24 h after stimulation with 1 *μ*g/mL LPS. Cells pretreated with Zuonin B displayed concentration-dependent inhibition of iNOS protein expression following LPS stimulation for 24 h. As shown in Figures 2(b) and 2(c), COX-2 protein was detected in untreated cells, and levels increased markedly after treatment with 1 *μ*g/mL LPS for 24 h, compared with the negative control (NC). Cells pretreated with Zuonin B displayed concentration-dependent inhibition of COX-2 protein expression following LPS stimulation for 24 h.

### 3.4. Effect of Zuonin B on NF-*κ*B and AP-1 Translocation

**Figure 3:**
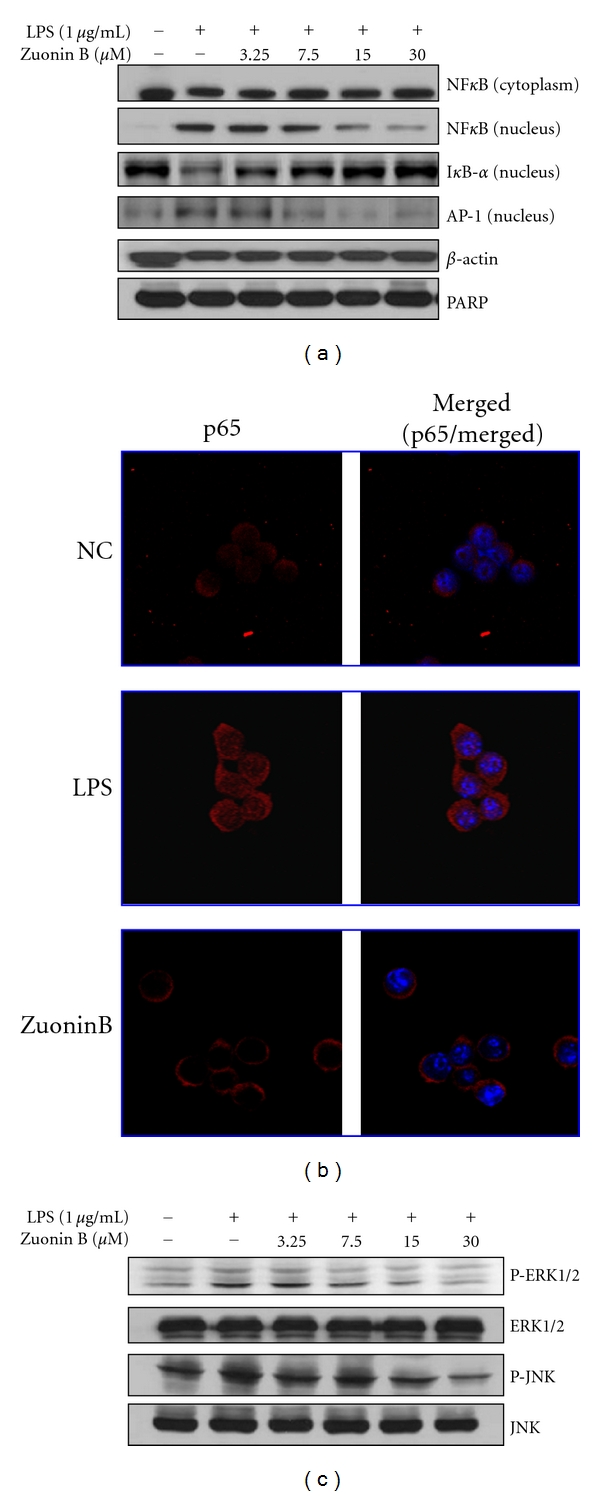
Effects of Zuonin B on NF-*κ*B activation and MAPK expression. (a) RAW264.7 cells were pretreated with different concentrations of Zuonin B for 1 h and stimulated with LPS (1 *μ*g/mL) for another 1 h. Equal amounts of protein in the cell lysates were electrophoresed, and the levels of NF-*κ*B (p65) protein in the cytosol and nucleus determined using specific antibodies for p65 and I*κ*B-*α*. The respective protein levels of *β*-actin and PARP were used to confirm equal amounts of protein for electrophoresis. (b) Immunofluorescence staining for NF-*κ*B p65. RAW264.7 cells were pretreated with 30 *μ*M Zuonin B for 1 h, followed by stimulation with LPS for another 1 h. Cells were fixed, permeabilized, and incubated with a specific antibody for NF-*κ*B p65, followed by Texas-red conjugated anti-rabbit Ig (red). The nuclei of the corresponding cells were visualized by DAPI staining (magnification  ×400). (c) Zuonin B inhibited LPS-stimulated phosphorylation of ERK1/2 and JNK in Raw264.7 cells. Cells were pretreated with different concentrations of Zuonin B for 1 h, and stimulated with LPS (1 *μ*g/mL) for another 30 min. NC: untreated control cells; LPS: LPS only treatment.

We further investigated whether Zuonin B prevented the translocation of the p65 subunit of NF-*κ*B from the cytosol to nucleus following release from I*κ*B-*α*, leading to induction of both iNOS and COX-2, with the aid of immunofluorescence staining. Nuclear and cytosolic extracts were subjected to immunoblot analysis. PARP (nuclear protein) and *β*-actin (cytosolic protein) were employed as controls to confirm the absence of contamination during extraction of each fraction. Our data show that p65 is distributed in the cytoplasmic compartment prior to LPS stimulation, but accumulates in the nucleus after LPS treatment. The p65 and AP-1 level in the nuclear fraction was significantly reduced upon pretreatment with Zuonin B (Figure 3(a)). As shown in Figure 3(a), Zuonin B inhibited degradation of I*κ*B-*α* as well as the LPS-induced increase in p65 in the nuclear fraction, indicating that the Zuonin-B-mediated suppression of I*κ*B-*α* degradationprevents NF-*κ*B-regulated expression. Immunofluorescence analyses revealed that in unstimulated cells, NF-*κ*B p65 was mainly present in the cytoplasm. After LPS treatment, the majority of intracellular p65 translocated from the cytoplasm to the nucleus, as evident from the strong nuclear NF-*κ*B p65 staining (Figure 3(b)).

### 3.5. Effects of Zuonin B on ERK1/2 and JNK Activation

Since the MAPK pathway is important for NF-*κ*B activation, we investigated whether MAPKs and NF-*κ*B are involved in Zuonin B-induced signaling in Raw264.7 cells. MAPK activation requires phosphorylation, detected using anti-phospho-MAPK and anti-MAPK antibodies specific for ERK1/2 and JNK. As shown in Figure 3(c), LPS induced phosphorylation of ERK1/2 and JNK in nontreated cells, whereas pretreatment with Zuonin B suppressed LPS-induced MAPK phosphorylation in a dose-dependent manner. Our results clearly indicate that Zuonin B inhibits LPS-induced NF-*κ*B activation via suppression of MAPK signaling.

### 3.6. Effects of Zuonin B on TNF-*α* and IL-6 Production

**Figure 4:**
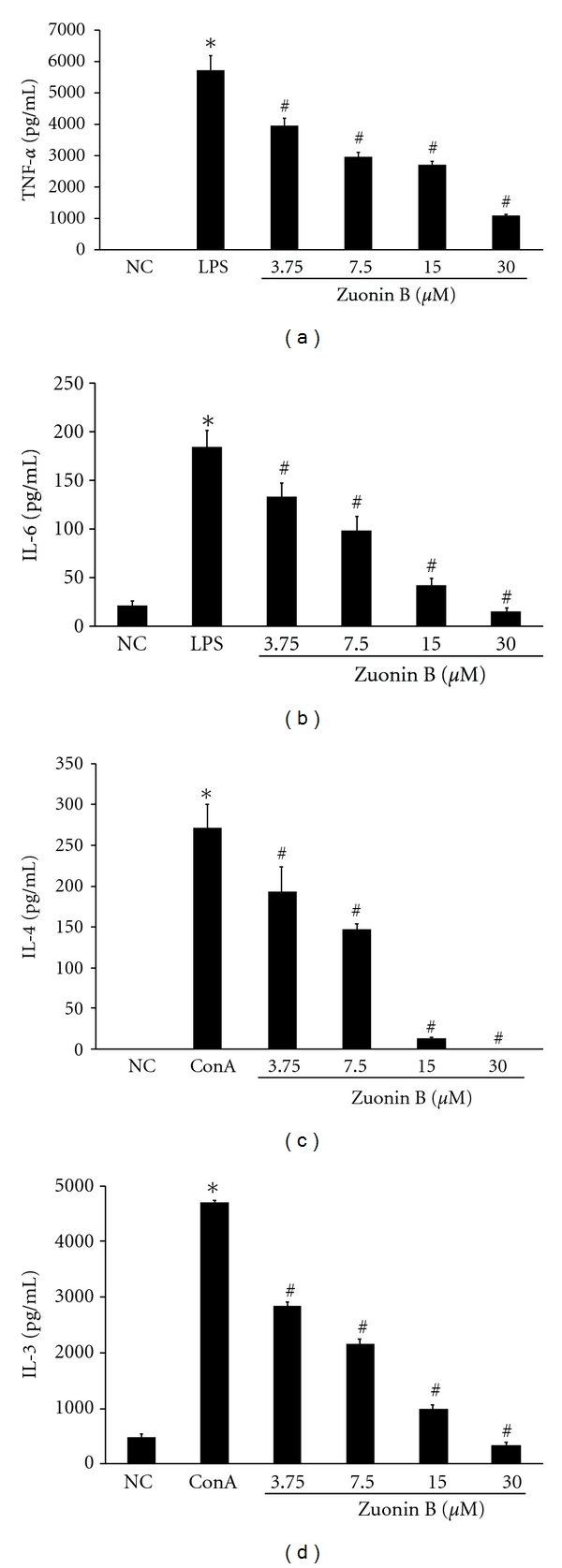
Effect of Zuonin B on Th1 (TNF-*α* and IL-6) and Th2 (IL-4 and IL-13) type cytokine production in LPS-stimulated RAW264.7 cells and ConA-induced splenocytes. RAW264.7 cells were pretreated with different concentrations of Zuonin B for 1 h and stimulated with LPS (1 *μ*g/mL) for another 24 h. Following incubation, the amounts of TNF-*α* (a) and IL-6 (b) released were determined with a TNF-*α* and IL-6 antibody-coated ELISA kit, as described in Section 2. Data represent the mean values of three experiments ± SEM. NC: untreated control cells; LPS: LPS only treatment.*Significant difference from NC and ^#^significant difference from LPS: *P* < 0.05. Con-A-stimulated splenocytes were cultured in the presence or absence of Zuonin B. Cytokine levels were measured using ELISA. Cytokine levels, IL-4 (c) and IL-13 (d). NC: untreated control cells; ConA, ConA (1 *μ*g/mL) only treatment. Data represent the mean values of three experiments ± SEM. *Significant difference from NC and ^#^significant difference from ConA, *P* < 0.05.

To confirm the inhibition of proinflammatory cytokines, TNF-**α** and IL-6 levels were additionally assessed using specific ELISA kits. As expected, pretreatment with Zuonin Bsignificantly inhibited TNF-**α**(Figure 4(a)) and IL-6 (Figure 4(b)) production in LPS-induced RAW264.7 cells.

### 3.7. Effects of Zuonin B on Th2-Type Cytokines in Splenocytes

Next, we examined the effects of Zuonin B on the production of cytokines (IL-4 and IL-13) in splenocytes. Treatment with ConA (1 *μ*g/mL) markedly enhanced IL-4 and IL-13 production in splenocytes. The results showed that ConA-stimulated splenocytessecretion of Th2type cytokines, such as IL-4 (Figure 4(c)) and IL-13 (Figure 4(d)), were inhibited by Zuonin Bin a concentration-dependent manner, compared with the controls.

## 4. Discussion

Inflammation is a critical factor in tumor progression. In this study, we investigated the effects of Zuonin B initially isolated from *Machilus thunbergii *on LPS-induced iNOS and COX-2 expression and its mode of action in RAW264.7 cells. Recent studies have shown that inflammation of these tissues is accompanied by upregulation of the inducible NO and iNOS isoforms [13]. The iNOS level is significantly correlated with the degree of inflammation [14]. Therefore, inhibitory effects against overproduction of NO and iNOS may provide a measure for assessing the anti-inflammatory effects of drugs on the anti-inflammatory process. In our experiments, Zuonin B inhibited NO production in a dose-dependent manner via suppression of iNOS protein expression in LPS-stimulated RAW264.7 cells. Based on these results, we suggest that Zuonin B may effectively relieve the inflammatory pathological process associated with excessive NO production.

PGE_2_ is an inflammatory mediator generated at inflammatory sites by COX-2, known as prostaglandin endoperoxidesynthase, that triggers the development of several chronic inflammatory diseases, such as cardiovascular disease, cancer, and rheumatoid arthritis [15]. COX-2, an inducible form of cyclooxygenase, serves as an interface between inflammation and cancer. In response to various stimuli, including bacterial LPS, COX-2 is transiently elevated in certain tissues. Abnormally elevated COX-2 causes promotion of cellular proliferation, suppression of apoptosis, enhancement of angiogenesis, and invasiveness, which account for its oncogenic function [16]. Hence, PGE_2_ and COX-2 are believed to be the target enzymes for anti-inflammatory activity. In our study, Zuonin B dose-dependently inhibited PEG_2_ production via suppressing COX-2 protein expression in LPS-stimulated RAW264.7 cells. These results indicate that Zuonin B is effective in COX-2-related inflammatory responses. 

Additionally, recent studies reveal that natural productsinhibit LPS-induced iNOS and COX-2 expression as well as TNF-*α* release in RAW264.7 macrophages by preventing NF-*κ*B and MAPK activation. In our experiments, Zuonin B inhibited LPS-induced TNF-*α* and IL-6 production. These findings suggest that Zuonin B exerts anti-inflammatory effects by inhibiting the secretion of proinflammatory cytokines. This compound is predominantly responsible for NF-*κ*B activation in response to proinflammatory stimuli [17]. NF-*κ*B and AP-1 are strong proinflammatory transcription factors, which can regulate a variety of inflammatory genes, including TNF-*α* [18]. NF-*κ*B is essential for host responses to microbial and viral infections, since the expression levels of several inflammation-related genes are regulated through the NF-*κ*B signaling pathway [19]. Our data indicate that Zuonin B inhibits the nuclear translocation of p65 protein via suppressing I*κ*B-*α* degradation, providing strong evidence that Zuonin B inhibits NF-*κ*B activation.

MAPKs involved in macrophage inflammation play important regulatory roles in cell growth and differentiation and control cellular responses to inflammatory cytokines and stress as well as NF-*κ*B activity [20]. Moreover, MAPKs play a central role in inducing cytokine production and mediating the cellular stress response [21, 22]. Several natural products inhibit the expression of these genes by modulating MAPK phosphorylation. In the current study, LPS induced rapid phosphorylation of ERK1/2 and JNK in RAW264.7 cells in the absence of Zuonin B. However, the precise signaling pathways among the three types of MAPKs are currently unclear. Zuonin B also diminished IL-4 and IL-13 production in a concentration-dependent manner in splenocytes. These results suggest that Zuonin B, at least in ConA-stimulated splenocytes, exerts the anti-inflammatory effects by suppressing the expression of proinflammatory enzymes as well as the secretion of proinflammatory cytokines.

In conclusion, Zuonin B exerts anti-inflammatory effects by suppressing intracellular NF-*κ*B activation, which leads to downregulation of the expression of inflammation-related proteins. In view of these results, we propose that the utility range of Zuonin B can be expanded as an anti-inflammatory therapeutic agent.
